# Effects of a nurse-led eHealth programme on functional outcomes and quality of life of patients with stroke: a pooled analysis of randomized controlled trials

**DOI:** 10.3389/fpubh.2024.1395270

**Published:** 2024-04-26

**Authors:** Wei Zhang, Zubing Mei, Zaibang Feng, Bin Li

**Affiliations:** ^1^Department of Neurology, Xinxiang Central Hospital, The Fourth Affiliated Hospital of Xinxiang Medical College, Xinxiang, China; ^2^Department of Anorectal Surgery, Shuguang Hospital, Shanghai University of Traditional Chinese Medicine, Shanghai, China; ^3^Anorectal Disease Institute of Shuguang Hospital, Shanghai, China; ^4^Department of Rehabilitation Medicine and Physiotherapy, Changhai Hospital, Naval Military Medical University, Shanghai, China; ^5^Department of Neurosurgery, Tongji Hospital, Tongji University School of Medicine, Shanghai, China

**Keywords:** stroke rehabilitation, eHealth interventions, nurse-led healthcare, randomized controlled trials (RCTs), functional outcomes, quality of life

## Abstract

**Background:**

Stroke remains a leading cause of disability worldwide. Nurse-led eHealth programs have emerged as a potentially effective strategy to improve functional outcomes and quality of life in stroke survivors. However, the variability of study designs and outcomes measured across trials necessitates a pooled analysis to comprehensively assess the efficacy of these interventions. This protocol outlines the methodology for a pooled analysis that aims to synthesize evidence from randomized controlled trials (RCTs) evaluating nurse-led eHealth interventions for stroke patients.

**Methods and analysis:**

This pooled analysis will be conducted according to the PRISMA guidelines. We will include RCTs that evaluate nurse-led eHealth programs and report on functional outcomes or quality of life in stroke patients. Comprehensive searches of electronic databases including Pubmed, EMBASE, the Cochrane Library, CINAHL, and PsycINFO will be conducted with a predefined search strategy. Study selection will involve screening titles and abstracts, followed by full-text review using explicit inclusion and exclusion criteria. Data extraction will be undertaken independently by two reviewers. The risk of bias will be assessed through the Cochrane Risk of Bias tool. Additionally, the quality of evidence for each outcome will be evaluated using the GRADE approach. Meta-analyses will be performed using random-effects models, and heterogeneity will be quantified using the I^2^ statistic. Subgroup and sensitivity analyses will explore potential sources of heterogeneity.

**Discussion and conclusions:**

This pooled analysis is poised to provide a nuanced understanding of the effectiveness of nurse-led eHealth programs in stroke rehabilitation, leveraging a thorough methodological framework and GRADE tool to ensure robustness and reliability of evidence. The investigation anticipates diverse improvements in patient outcomes, underscoring the potential of personalized, accessible eHealth interventions to enhance patient engagement and treatment adherence. Despite the challenges posed by the heterogeneity of interventions and rapid technological advancements, the findings stand to influence clinical pathways by integrating eHealth into standard care, if substantiated by the evidence. Our study’s depth and methodological rigor possess the potential to initiate changes in healthcare policy, advocating for the adoption of eHealth and subsequent investigations into its cost-efficiency. Ultimately, we aim to contribute rich, evidence-based insights into the burgeoning field of digital health, offering a foundational assessment of its applications in stroke care. Our data is expected to have a lasting impact, not only guiding immediate clinical decisions but also shaping the trajectory of future healthcare strategies in stroke recovery.

**Systematic review registration:**

Identifier (CRD42024520100: https://www.crd.york.ac.uk/prospero/display_record.php?RecordID=520100).

## Introduction

Stroke remains a leading cause of long-term disability worldwide, with significant impacts on physical function, cognitive abilities, and quality of life (1). Rapid advancements in technology have led to the burgeoning field of eHealth, defined as the use of information and communication technologies for health (2). These digital solutions have been increasingly integrated into rehabilitation practices for stroke survivors. The potential of eHealth programs is vast, offering accessible care and enhanced patient engagement, especially when led by healthcare providers such as nurses (3).

The importance of a comprehensive stroke rehabilitation strategy that incorporates nurse-led eHealth programs cannot be overstated. Nurses play a crucial role in patient education, long-term care, and monitoring progress, which are all critical in the post-stroke recovery phase (4). Nurse-led interventions are particularly promising, as they leverage the nexus of clinical expertise and patient-centered care within the digital health paradigm (5).

The global healthcare landscape is also experiencing a shift from traditional in-person care to telehealth and remote monitoring systems (6). In stroke care, eHealth interventions present a unique opportunity to extend the reach of rehabilitation services, providing continuity of care that transcends the bounds of hospital settings (7). In China, where the burden of stroke is also considerable and climbing, investing in innovative healthcare delivery methods such as eHealth programs becomes not only advantageous but essential. The healthcare system in China, with its diverse and populous landscape, presents both challenges and opportunities for the management of chronic conditions like stroke. Recognizing this, nurse-led eHealth interventions are primed to play a pivotal role, which can offer a potent combination of technology and personalized care. Randomized controlled trials (RCTs) remain the gold standard for evaluating the effectiveness of these interventions (8), and meta-analyses of such trials provide high-level evidence, synthesizing results for clearer clinical insights (9).

A significant body of literature has emerged, highlighting the efficacy of eHealth solutions in managing chronic diseases and facilitating rehabilitation (10). For instance, tele-rehabilitation has been explored as a viable option to support stroke patients in rural and underserved areas (11). Furthermore, eHealth interventions enable personalized care, adapting to the unique needs and recovery pace of each patient (12).

Nurse-led eHealth programs have the potential to significantly impact patients’ functional outcomes and quality of life through structured, technology-assisted interventions. Studies have shown that such programs can lead to improvements in self-management, physical activity, and adherence to rehabilitation regimes (13, 14). However, the evidence is fragmented, and a systematic evaluation of the available data is needed to understand the true impact of these interventions.

Given the multidimensional nature of stroke recovery, incorporating eHealth programs into routine care could offer a scalable and effective solution to meet the diverse needs of stroke survivors (7). With the increasing prevalence of stroke and the subsequent demand for rehabilitation services, nurse-led eHealth interventions present a compelling solution for healthcare systems strained by a growing patient population (15).

Effective stroke rehabilitation requires a holistic approach that encompasses not just physical recovery but also psychological support, lifestyle coaching, and social reintegration (16, 17). Nurse-led eHealth programs are strategically positioned to deliver such comprehensive care, leveraging the rapport nurses maintain with patients to drive meaningful engagement and ultimately, promote better health outcomes (18).

As healthcare moves towards more patient-centered models, the role of nurses in delivering and coordinating eHealth interventions will likely become more prominent (19). By integrating digital tools with clinical expertise, nurses can orchestrate personalized care plans, monitor patient progress remotely, and adjust interventions in real-time based on data-driven insights (20).

Given these considerations, this study aims to synthesize evidence on the efficacy of nurse-led eHealth programs in enhancing functional outcomes and improving the quality of life for stroke survivors. The study will provide valuable guidance for clinicians, healthcare policymakers, and stakeholders on the integration of digital health solutions into stroke rehabilitation practices.

## Methods

In our pooled analysis, we will rigorously adhere to the Preferred Reporting Items for Systematic Reviews and Meta-analyses (PRISMA) guidelines.

### Study registration

This study has been registered in International prospective register of systematic reviews (PROSPERO) website, an international database of prospectively registered studies or reviews, obtaining a PROSPERO preregistration number of CRD42024520100. Registration ensures transparency, reduces duplication, and prevents reporting bias by pre-specifying the research questions, methodology, and analysis strategies (21). This process provides an up-to-date record of all systematic reviews in progress, which contributes to the integrity of the systematic review methodology (22, 23).

### Eligibility criteria

We will include studies that assess the impact of nurse-led eHealth interventions compared to usual care or other interventions on functional outcomes and quality of life on stroke patients, with the following specifications for participants’ baseline characteristics:

1. Severity of stroke: Studies must classify the severity of stroke using a recognized scale such as the National Institutes of Health Stroke Scale (NIHSS) or the modified Rankin Scale (mRS).
2. Time since onset: Studies should enroll participants within a specific timeframe post-stroke (e.g., within 6 months from onset).
3. Comorbidities: Studies must provide a detailed description of participants’ comorbid conditions to ensure that any additional health issues are accounted for in the analysis.

Studies will be selected based on pre-defined inclusion and exclusion criteria, including peer-reviewed publications, language restrictions to English, and a publication date after the year 2000. Additionally, only studies that report quantifiable outcomes relevant to stroke rehabilitation will be considered.

### Participants

The participants will be adults aged 18 and over who have experienced a stroke, as defined by the World Health Organization (24). Studies targeting both early and late post-stroke phases will be included, without any restrictions based on the type of stroke (ischemic or hemorrhagic), to generalize findings across a broad stroke population.

### Interventions

Studies that evaluate the impact of eHealth interventions led by nurses will be included. These interventions may encompass tele-rehabilitation, mobile health apps, virtual reality, remote monitoring, or any form of digital health technology utilized to assist in stroke rehabilitation (25, 26). The interventions must be primarily managed or facilitated by nursing professionals to qualify for inclusion.

### Comparisons

The primary comparisons will be between nurse-led eHealth interventions and standard care practices or alternative rehabilitation strategies. Both active and passive comparators will be included, given that they provide context on the effectiveness of the interventions under review.

### Outcomes

Primary outcomes will include functional independence, assessed by the Barthel Index (27) and the Modified Rankin Scale (28), and quality of life, measured using the Stroke-Specific Quality of Life Scale (29). Secondary outcomes will span depression and anxiety levels, determined by the Hospital Anxiety and Depression Scale (30); rehospitalization rates, indicative of the intervention’s success in maintaining health stability; patient satisfaction with the eHealth programme; adherence to programme protocols; and a cost-effectiveness analysis comparing conventional care (31).

Adherence will be defined as the percentage of the prescribed eHealth intervention sessions completed by participants. The threshold for satisfactory adherence will be set at 80% completion, consistent across all studies. Suitable adherence measures including self-reported logs, electronic health record audits, and programme usage data, will be synthesized from included studies. A comprehensive cost-effectiveness analysis of nurse-led eHealth interventions for stroke rehabilitation involves the specific cost components, which includes initial setup costs (encompassing the expenses associated with implementing eHealth technologies, including hardware, software, and personnel training), maintenance costs (accounted for ongoing charges for software updates, technical support, and hardware servicing), patient-related costs (costs borne by the patients, such as travel for face-to-face assessment, technology usage costs, and any informal care expenses), and healthcare resource utilization (the cost of medical support, therapy sessions, and additional healthcare services necessitated by the eHealth intervention). These comprehensive metrics will deliver insights into the multifaceted impacts of the eHealth intervention on stroke recovery.

### Study design

The proposed study will adopt a pooled analysis design, encompassing a systematic aggregation and synthesis of data from multiple RCTs examining the influence of nurse-led eHealth programs on post-stroke rehabilitation. This method will enable a robust assessment of interventional efficacy across diverse populations and healthcare settings. Through this meta-analytical approach, we aim to generate high-quality evidence regarding the outcomes of functional independence and quality of life in patients who have experienced a stroke.

### Search strategy and identification of studies

#### Literature searches

A comprehensive search strategy will be developed and applied to multiple databases including Pubmed, EMBASE, the Cochrane Library, CINAHL, and PsycINFO to identify studies relevant to the research question from the inception to March, 2024. The search strategy will incorporate a combination of MeSH terms and keywords relevant to stroke, rehabilitation, eHealth, and nursing interventions, without language restrictions (Table 1). The search strategy will be peer-reviewed by an information specialist using the PRESS checklist (32). We will also implement systematic searches of clinical trial registries, conference proceedings, and directly contact authors of unpublished studies related to nurse-led eHealth programs for stroke rehabilitation, ensuring a more inclusive and exhaustive synthesis of available evidence.

**Table 1:** Search strategy for Pubmed database.

#	Search terms
1	stroke[MeSH Terms] OR cerebrovascular accident OR cerebral infarction OR brain vascular accident
2	eHealth[MeSH Terms] OR eHealth OR telemedicine OR telehealth OR digital health OR mobile health
3	nurse-led[Title/Abstract] OR nursing intervention[MeSH Terms] OR nurse-administered
4	functional outcomes[MeSH Terms] OR functional recovery OR activity of daily living OR rehabilitation outcomes OR quality of life[MeSH Terms] OR life quality OR health-related quality of life
5	randomized controlled trial[Publication Type] OR RCT OR controlled clinical trial[MeSH Terms]
6	#1 AND #2 AND #3 AND #4 AND #5

#### Study selection

Study selection will be a two-phase process. Initially, following the removal of duplicates, two independent reviewers will screen titles and abstracts against the inclusion criteria. Full-text articles will then be obtained for further assessment. Disagreements will be resolved through discussion or consultation with a third reviewer if necessary.

#### Data extraction

Once the studies are identified, two reviewers will independently extract relevant data from the included RCTs using a structured data extraction form. Key information to be extracted includes study design, sample size, participant demographics, details of the nurse-led eHealth intervention (including duration, frequency, components, and technology used), comparators, outcome measures (specifically, scales for functional outcomes such as the Barthel Index, and quality of life assessments like the Stroke-Specific Quality of Life Scale), time points of outcome measurement, and results (including effect sizes and confidence intervals). Discrepancies between reviewers will be resolved through discussion or by consulting a third reviewer. Data extraction forms will be pre-piloted to ensure consistency in the gathered information.

### Risk of bias assessment

Risk of bias assessment will be systematically conducted for each included study using the Cochrane Collaboration’s tool for evaluating the risk of bias in randomized trials. Key domains to be appraised include random sequence generation, allocation concealment, blinding of participants and personnel, blinding of outcome assessment, incomplete outcome data, selective reporting, and other sources of bias (33). Each domain will be judiciously judged as “low risk,” “high risk,” or “unclear risk” of bias, with justifications documented. Any disagreements during the assessment will be resolved through discussion or involvement of a third reviewer. The overall risk of bias for each study will be incorporated in the analysis to assess the strength of the evidence and potential impact on the study’s findings.

### Quality of evidence assessment

The quality of evidence for each outcome will be assessed using the Grading of Recommendations Assessment, Development, and Evaluation (GRADE) approach. This tool appraises the quality of evidence based on several criteria, including the risk of bias, inconsistency, indirectness, imprecision, and publication bias. Evidence quality is categorized into four levels: high, moderate, low, or very low (34). These ratings will inform the strength of our recommendations and the degree of confidence in the effect estimates. Application of the GRADE framework will be carried out by two independent reviewers, with discrepancies resolved through consensus or arbitration by a third reviewer.

### Data synthesis and statistical analysis

We will deploy STATA software (version 16) to synthesize data from multiple trials. Continuous outcomes, such as functional independence operationalized by the Barthel Index and subjective well-being as measured via the Stroke-Specific Quality of Life Scale, will be amalgamated using the inverse variance method, yielding weighted mean differences (WMDs). Dichotomous data, such as incidence of rehospitalization, will be consolidated employing a Mantel–Haenszel approach to calculating risk ratios (RRs). Dependent on the extent of inter-study heterogeneity, as ascertained by an I^2^ statistic threshold of 50%, data will be interpreted through a random-effects model to accommodate variability (35).

To explore potential sources of heterogeneity and to ascertain the influence of varying interventions and patient variables on the outcomes, our subgroup analyses will include the following dimensions:

1. Types of eHealth interventions: We will categorize interventions into subgroups such as tele-rehabilitation, mobile health (mHealth), and electronically delivered patient education.
2. Duration of treatment: Given that the length of intervention may impact the effect size, subgroups will be defined based on short-term (<3 months), medium-term (3–6 months), and long-term (>6 months) interventions.
3. Variation in stroke severity: Recognizing that initial stroke severity can affect recovery and adaptation to eHealth interventions, studies will be subgrouped according to the stroke severity assessment scales (NIHSS or mRS scores) utilized.
4. Additional heterogeneity factors such as frequency of contact (e.g., daily, weekly, monthly), technological features (e.g., mobile app, web-based platform, telehealth), and cultural context (e.g., urban versus rural settings, cultural tailoring of the intervention) will be examined.

Publication bias will be assessed if ten or more studies are included in the meta-analysis. Our protocol will involve the following two steps to detect and mitigate potential publication bias including visual examination of funnel plots and implementation of Egger’s test (36). Firstly, we will generate funnel plots of the effect sizes versus the standard error for each included study to visually assess asymmetry, which can be indicative of publication bias. In addition to the visual assessment, we will use Egger’s test for statistically evaluating funnel plot asymmetry to quantify the likelihood and direction of any potential publication bias. Should publication bias be detected, the “trim and fill” method will be utilized to adjust for resulting asymmetries (37).

### Update plan

In recognition of the rapid advancements in eHealth technologies, we have developed a proactive update strategy to ensure that our study remains current and reflective of best practices. Specifically, we will include the following measures:

Firstly, we will establish a cut-off date for study inclusion, set at six months prior to our anticipated review completion date. This approach enables the inclusion of the most recent trials and mitigates the risks associated with potential obsolescence of eHealth applications. Secondly, to maintain the review’s relevance amidst technological changes, we commit to conducting biennial updates. These updates will incorporate new data from recent trials and ensure our conclusions align with the latest eHealth developments. Thirdly, we plan to monitor ongoing trials and maintain a comprehensive database of upcoming studies through clinical trial registries and ongoing dialogue with domain experts and researchers. This monitoring will inform our biennial review updates. Fourthly, our update strategy includes flexibility to adapt to significant innovations and developments in the eHealth landscape. Should emergent technologies redefine best practices, we will adjust our review scope and analysis accordingly. Lastly, updated findings will be disseminated promptly through publication addendums, ensuring that healthcare providers, researchers, and policymakers have access to the most current evidence.

### Patient and public involvement

In this study, the engagement of patients and the general public will not occur, as the methodology consists solely of pooled data analysis from published trials which does not include direct patient or public involvement. Nevertheless, once the study is concluded, we will actively distribute our findings and ensure that the outcomes are communicated via various social media platforms.

### Ethical considerations

Ethical approval is not typically required for this study as they are based on published data. However, all efforts will be made to handle data responsibly and maintain confidentiality and privacy.

### Amendments

Any amendments to the protocol will be documented with reference to the date of the change and the specific changes made. This ensures transparency in the research methodology.

## Discussion

The potential findings of our study will likely center around the efficacy and optimal application of stroke rehabilitation through eHealth platforms. Given the increasing incorporation of eHealth in post-stroke care, it is anticipated that studies may demonstrate varied levels of improvement in patient outcomes, from motor function to quality of life (4).

The mechanisms behind these findings may be attributed to the increased accessibility and personalized nature of eHealth interventions, which can lead to higher patient engagement and adherence to rehabilitation protocols. The advantages of eHealth interventions may lie in their capability to offer patients remote access to rehabilitation programs, which is particularly beneficial for stroke survivors who often face mobility and transportation challenges. Additionally, the personalized nature of eHealth programs allows for individual tailoring of interventions to specific patient needs and recovery goals. This customization is paramount for stroke patients, given the diverse ways this condition impacts individuals. Our findings may suggest that such personalization, facilitated by nurse-led interventions, improves patient engagement by providing a sense of agency in the rehabilitation process and encouraging adherence to prescribed routines. Through remote monitoring and feedback, eHealth platforms can enable more frequent patient-provider interactions, fostering a supportive environment that positively influences adherence to rehabilitation protocols. Hence, the relevance of these mechanisms in this study may be their direct impact on overcoming barriers to effective rehabilitation in stroke survivors, leading to improved functional outcomes and quality of life.

Strengths of our study include a rigorous methodological approach to search strategy and selection criteria, which are aimed at minimizing bias and maximizing the comprehensiveness of the literature synthesized. The adoption of the GRADE tool in assessing the quality of evidence further reinforces the reliability of our findings both for clinicians and policymakers (38).

However, limitations are also present. The heterogeneity in eHealth interventions and patient populations may complicate the synthesis of data and impact the ability to draw generalizable conclusions. Additionally, in consideration of the rapid pace at which eHealth technologies advance, we acknowledge that the eHealth applications assessed in our pooled analysis may evolve significantly between the completion of the included trials and the publication of this study. It highlights the need for ongoing evaluation of eHealth solutions to ensure that our analysis reflects the most current and clinically applicable technologies. To address the potential risk of obsolescence, we emphasize the importance of aligning eHealth interventions with prevailing technological standards and calls for continual updates such evidence in clinical practice.

From a clinical perspective, the findings could shape future rehabilitation strategies by emphasizing the benefits of integrating eHealth solutions into standard care. If the evidence supports eHealth as an effective modality for stroke rehabilitation, it could lead to wider adoption amongst healthcare providers and inform patient care pathways (39).

For policymakers, the impact of our findings could be substantial, suggesting potential for eHealth to improve patient outcomes, potentially at a lower cost compared to traditional rehabilitation methods. This could drive policy changes that support the implementation of eHealth interventions within public healthcare systems, and prompt further research into the cost-effectiveness of such interventions (40).

In conclusion, this study is expected to add valuable insights into the field of stroke rehabilitation, explicating the role of eHealth in improving patient outcomes. While acknowledging the limitations of the study, the strengths and the breadth of the evidence evaluated promise to provide a sound basis for both clinical applications and policy considerations, ultimately contributing to better healthcare delivery for stroke survivors.

## Author contributions

WZ: Writing – review & editing, Writing – original draft, Validation, Resources, Methodology, Investigation, Conceptualization. ZM: Writing – review & editing, Writing – original draft, Validation, Supervision, Methodology, Conceptualization. ZF: Writing – review & editing, Writing – original draft, Validation, Methodology, Conceptualization. BL: Writing – review & editing, Writing – original draft, Validation, Supervision, Resources, Investigation.
